# Identification and characterization of GH11 xylanase and GH43 xylosidase from the chytridiomycetous fungus, *Rhizophlyctis rosea*

**DOI:** 10.1007/s00253-018-9431-5

**Published:** 2018-11-05

**Authors:** Yuhong Huang, Xianliang Zheng, Bo Pilgaard, Jesper Holck, Jan Muschiol, Shengying Li, Lene Lange

**Affiliations:** 1 Biotechnology and Biomedicine, Technical University of Denmark, 2800 Kgs. Lyngby, Denmark; 2 Sino-Danish Center for Education and Research, Beijing, 100190 China; 3 Present Address: The National Food Institute, Technical University of Denmark, Building 201, Søltofts Plads, 2800 Kongens Lyngby, Denmark; 4 Qingdao Institute of Bioenergy and Bioprocess Technology, Chinese Academy of Sciences, Qingdao, 266101 China; 5 Chemical and Biochemical Engineering, Technical University of Denmark, 2800 Kgs. Lyngby, Denmark

**Keywords:** *Rhizophlyctis rosea*, *Chytridiomycota*, HotPep, GH11 xylanase, GH43 xylosidase

## Abstract

The early-lineage, aerobic, zoosporic fungi from the *Chytridiomycota* constitute less than 1% of the described fungi and can use diverse sources of nutrition from plant or animal products. One of the ancestral sources of fungal nutrition could be products following enzymatic degradation of plant material. However, carbohydrate-active enzymes from these ancient fungi have been less studied. A GH11 xylanase (RrXyn11A) (EC 3.2.1.8) and a GH43 xylosidase (RrXyl43A) (EC 3.2.1.37) were identified from an early-lineage aerobic zoosporic fungus, *Rhizophlyctis rosea* NBRC 105426. Both genes were heterologously expressed in *Pichia pastoris* and the recombinant enzymes were purified and characterized. The optimal pH for recombinant RrXyn11A and RrXyl43A was pH 7. RrXyn11A had high stability over a wide range of pH (4–8) and temperature (25–70 °C). RrXyn11A also showed high substrate specificity on both azurine-cross-linked (AZCL) arabinoxylan and AZCL xylan. RrXyl43A had β-xylosidase and minor α-l-arabinofuranosidase activity. This enzyme showed low product inhibition and retained 51% activity in the presence of 100 mM xylose. A combination of RrXyn11A and RrXyl43A exhibited significantly higher hydrolytic and polymer degradation capability and xylose release on wheat bran and beechwood xylan compared to treatment with commercial enzymes. This study was the first to heterologously express and characterize the GH11 xylanase (RrXyn11A) and GH43 xylosidase (RrXyl43A) from the ancient fungus, *R. rosea*. Meanwhile, this study also demonstrated that the enzymes from the ancient fungus *R. rosea* can be easily handled and heterologously expressed in *Pichia*, which presents a promising path to a new source of enzymes for biomass degradation.

**Electronic supplementary material:**

The online version of this article (10.1007/s00253-018-9431-5) contains supplementary material, which is available to authorized users.

## Introduction

*Rhizophlyctis rosea* (*Rhizophlyctidales*, *Chytridiomycota*) is an early-lineage aerobic zoosporic fungus. These early-lineage fungi make up less than 1% of the described fungi and can use diverse sources of plant or animal origin as nutrition (Stajich et al. 2009). Products produced by breakdown of plant material may have been one of the original sources of nutrition for fungi (Chang et al. 2015). *R. rosea* can be found worldwide in agricultural soils and is especially easy to isolate from soil extract after rain (Willoughby 2001; Marano et al. 2011). Most chytrid research up until now has focused on studies of interactions with hosts and substrates and on the ecology and morphology of these early-lineage fungi (Stanier 1942; Chambers and Willoughby 1964; Willoughby 2001; Marano et al. 2011; Chang et al. 2015; Gleason et al. 2017). The majority of fungal carbohydrate-active enzymes described and used at present come from *Ascomycota*, *Basidiomycota*, and *Zygomycota* species (Zhao et al. 2013). However, the ancient fungi could also be potential candidates for discovery of enzymes for biomass conversion (Lange et al. 2018). *R. rosea* has been found to have cellulose degradation capability (Stanier 1942; Willoughby 2001). Yet until now, only one endoglucanase from the GH45 family from *R. rosea* has been heterologously expressed and characterized (Schulein et al. 2002; Pilgaard 2014; Lange et al. 2018).

Plant cell wall polysaccharides of lignocellulosic agricultural crop residues are the most abundant biomass components and provide an important resource for upgrading to high value-added products through biorefinery processes (Kamble and Jadhav 2012). Xylan is the major component of hemicellulose and represents around 20% of agricultural biomass depending on the origin (Scheller and Ulvskov 2010). It is a linear polysaccharide consisting of β-1,4-linked xylose units with a large variety of side-chain substituents, such as sugars (arabinose, xylose, galactose), glucuronic acids, and the acetyl, feruloyl, and *p*-coumaryl groups (Paës et al. 2012). The effective degradation of xylan is a key process for the conversion of hemicellulose-rich biomass to high value-added products. Several enzymes are required for complete hydrolysis of xylan (Mendis et al. 2016). Among them, xylanases and β-xylosidases play a crucial role in the hydrolysis of the xylan backbone. Xylanases from the GH11 family (EC 3.2.1.8) have some interesting properties such as high substrate specificity and catalytic efficiency when compared with other endo-β-1,4-xylanases from the GH5 (EC 3.2.1.8), GH8 (EC 3.2.1.8), GH10 (EC 3.2.1.8), GH30 (EC 3.2.1.8), and GH43 (EC 3.2.1.8) families (Paës et al. 2012). β-xylosidase can further hydrolyze the short oligomers of β-d-xylopyranosyl units that accumulate as a result of xylanase action. β-xylosidases are assigned to the GH3 (EC 3.2.1.37), GH39 (EC 3.2.1.37), GH43 (EC 3.2.1.37), GH52 (EC 3.2.1.37), and GH54 (EC 3.2.1.37) families. GH43 β-xylosidases have an inversion reaction mechanism which is different from other families (Manju and Singh Chadha 2011). Most GH43 β-xylosidases are derived from bacteria. Only a few GH43 β-xylosidases have been characterized from ascomycetous filamentous fungi such as *Penicillium oxalicum* (Ye et al. 2017), *Humicola insolens* (Yang et al. 2014), *Aspergillus oryzae* (Suzuki et al. 2010), and *Thermomyces lanuginosus* (Chen et al. 2012).

Peptide Pattern Recognition (PPR) is a non-alignment-based sequence analysis platform, which can identify short, conserved sequence motifs for the enzymes and is used for efficient prediction of the enzyme function from sequences (Busk and Lange 2013a; Busk et al. 2017). PPR-based HotPep was used to discover the plant cell wall-degrading enzymes in the genome of *R. rosea*. Two proteins were annotated from the genome of *R. rosea* as GH11 xylanase (RrXyn11A) (EC 3.2.1.8) and GH43 xylosidase (RrXyl43A) (EC 3.2.1.37). They were both heterologously expressed in *Pichia pastoris* KM71H. The purified recombinant enzymes were further characterized with respect to pH and temperature optimum and hydrolytic capability. Our study indicated that RrXyn11A and RrXyl43A from the early-lineage fungus *R. rosea* have promising potential for biomass conversion.

## Materials and methods

### Gene prediction and functional annotation

The genome of *R. rosea* Fischer, NBRC 105426 (GCA_002214945.1), was downloaded from GenBank and all putative protein sequences in the genome were predicted with the Augustus web server (http://bioinf.uni-greifswald.de/webaugustus/) (Stanke and Morgenstern 2005) using *Rhizopus oryzae*, which is the taxonomically closest organism in the list, as a model for prediction. The list of protein sequences was annotated with respect to carbohydrate-active enzymes using HotPep (Busk et al. 2017; Lange et al. 2018) and the dbCAN server (Huang et al. 2018). The two putative genes were submitted to GenBank and assigned temporary accession numbers. The GH11 protein sequence was named RrXyn11A (unmodified gene accession MF432128, codon-optimized gene accession MH476189) and the GH43 protein sequence was named RrXyl43A (unmodified gene accession MF432129, codon-optimized gene accession MH476190).

### Sequence and structure analysis

The signal peptide was predicted by SignalP 4.1 (Petersen et al. 2011). The conserved domain was analyzed by dbCAN server (Huang et al. 2018). The *N*-glycosylation site was predicted by NetNGlyc 1.0 Server (Gupta et al. 2004). 3D structure modeling of RrXyn11A and RrXyl43A was prepared using YASARA 16.9.23 (YASARA Biosciences 26 GmbH, Vienna, Austria) using the built-in homology modeling function; possible templates were identified by running three PSI-BLAST interactions to extract a position-specific scoring matrix (PSSM) from UniRef90, and then the PDB was searched for a match. For RrXyn11A and RrXyl43A, the structures 5GLR, 4MLG, 5A8C, 3C7F, and 4NOV and the structures 5GLR, 4MLG, 5A8C, 3C7F, and 4NOV, respectively, were automatically identified as templates for preparation of the homology models. After preparing five models for each template, YASARA combined the best parts of each model based on a quality ranking in a hybrid model. The resulting hybrid models were further refined using the md_refine macro as supplied with the YASARA package. The model with the lowest energy after refinement was quality checked using QMEAN4 (Benkert et al. 2008) and used for substrate superposition. The models were subsequently aligned in YASARA using the MUSTANG method (Konagurthu et al. 2006) to structures with co-crystalized ligands (4HK8 for the GH11 and 2EXJ for the GH43). Then the ligand from the crystal structure was joined to the homology model followed by an energy minimization step using the YASARA force field (Krieger et al. 2009). The Ara-(α1-3)-Xyl docked GH43 model was prepared by modifying the ligand in the xylobiose docked model. All models and substrate superposed structures were visualized in PyMOL (ThePyMOL Molecular Graphics System, Version 1.1 Schrödinger, Cambridge, MA, USA).

### Phylogenetic analysis

Accession numbers representing characterized enzymes were retrieved from the CAZy database (Lombard et al. 2014) (September 2017) and corresponding sequences were downloaded from GenBank (https://www.ncbi.nlm.nih.gov/genbank/) (Supplemental Fig. S1 and S2). The catalytic domains were predicted with dbCAN HMM models (Yin et al. 2012) in the HMMer3 package v3.1b2 (Eddy 2011) locally. The sequences were truncated to include only the predicted domains and aligned using MAFFT (Katoh and Standley 2013) in the PipeAlign2 server (Plewniak et al. 2003). In cases where two or more of the same catalytic domains were found in the same sequence, a letter starting from “-A” was appended to the accession number and the sequence included in the analysis. The multiple alignments were used to build LG maximum likelihood phylogenetic trees using RaxML-HPC BlackBox (v. 8.2.10) (Stamatakis et al. 2008) at the CIPRES science gateway (Miller et al. 2010) with 1000 bootstrap replications. The trees were visualized in ITOL (Letunic and Bork 2016).

### Heterologous expression of RrXyn11A and RrXyl43A

The cDNA sequences of RrXyn11A and RrXyl43A were codon-optimized according to yeast codon bias and further synthesized and cloned in pPICZαA by GenScript (Piscataway, USA). The plasmids were digested with restriction enzyme *Pme*I and transformed into *P. pastoris* KM71H by electroporation according to the EasySelect *Pichia* Expression kit manual (Invitrogen, Waltham, USA). YPDS plates containing 100 μg/ml zeocin were used for preliminary selection of the positive transformants harboring chromosomal integration of gene expression cassettes. YPDS plates supplemented with increasing concentration of zeocin (500–2000 μg/ml) were used for further screening of multi-copy integrated recombinants.

Small-scale and large-scale expressions of recombinant enzymes were performed according to the EasySelect *Pichia* Expression kit manual (Invitrogen, Waltham, USA). Supernatant was harvested by centrifugation at 1500*g* for 5 min at 4 °C and filtered through a 0.45-μm filter (Minisart Syringe Filters, Sartorius, Goettingen, DE) and then used for enzyme activity analysis. Supernatant from large-scale expression was further concentrated using VIVASPIN 20, 10,000 MWCO filter (Sartorius, Goettingen, DE) for purification.

### Purification of the recombinant enzymes

The recombinant proteins were purified by fast protein liquid chromatography (FPLC) on an ÄKTA purifier equipped with a 5-ml HisTrap™ HP crude affinity column (GE Healthcare, Freiburg, DE). First, the HisTrap™ HP column was equilibrated with binding buffer (20 mM sodium phosphate, 500 mM NaCl, 30 mM imidazole, pH 7.4) with a flow rate of 5 ml/min. Then 10 ml concentrated sample was loaded into the column which was further washed with binding buffer for four column volumes. Finally, the column was gradient eluted with elution buffer (20 mM sodium phosphate, 500 mM NaCl, 500 mM imidazole, pH 7.4). Purified enzymes were collected and the elution buffer was replaced with protein buffer (20 mM sodium phosphate, 100 mM NaCl, 10% glycerol, pH 7.4).

### Deglycosylation

Purified recombinant enzymes were deglycosylated by Endo H (P0702S, NEW ENGLAND BioLabs, Ipswich, UK) according to the following protocol. Sixty micrograms of purified recombinant RrXyn11A was combined with RrXyl43A and 3 μl of Glycoprotein Denaturing Buffer and heated at 100 °C for 10 min, then made up to a total reaction volume of 60 μl by adding 2 μl of 10× GlycoBuffer 3, 2 μl Endo H and H_2_O and incubated at 37 °C for 1 h.

### SDS-PAGE and western blot analysis

Crude, purified recombinant enzyme, or deglycosylated enzyme were mixed with 4 × Laemmli sample buffer (161-0747, Bio-Rad, Copenhagen, DK) and 500 mM 1,4-dithiothreitol. Then the samples were incubated at 99 °C for 10 min and loaded on the 12% Mini-PROTEAN® TGX™ Precast Protein Gels (4561045, Bio-Rad, Copenhagen, DK). The Precision Plus Protein™ Dual Color Standard (1610394, Bio-Rad, Copenhagen, DK) was included as marker. After electrophoresis, gels were washed three times with distilled water for 15 min and stained using Bio-Safe™ Coomassie Stain (1610787, Bio-Rad, Copenhagen, DK) for 1 h, followed by de-staining using distilled water for 2 h. Western blot was performed as follows. After SDS-PAGE electrophoresis, gel was soaked in 100 ml of electroblotting buffer (1 × Tris-Glycine buffer) for 5 min. PVDF membrane (Amersham HydondTM-LFP, Hybond LFP PVDF transfer membrane, RPN303LFP, GE Healthcare, Amersham, UK) was wetted in 96% ethanol for a few seconds and then transferred to electroblotting buffer. Sponges and filter paper (criterion™ Blotter Filter Paper, 1704085, Bio-Rad, Copenhagen, DK) were dipped in a separate container of electroblotting buffer. Sponge, filter paper, membrane, and gel were assembled and inserted in the blotting apparatus with electroblotting buffer. After the transformation was complete, blotting membrane was removed and rinsed with distilled water. The membrane was incubated in 50–100 ml 1 × TBS with 2% skimmed milk powder and 0.1% Tween 20 for 1 h, which followed by incubation in 1 × TBS with 2% skimmed milk powder, 0.1% Tween 20, and primary antibody (monoclonal Anti-polyHistidine peroxidase conjugated antibody (Sigma-Aldrich, Steinheim, DE), 1:10000 dilution) for 1 h. Then the membrane was washed with 1 × TBS with 2% skimmed milk powder and 0.1% Tween 20 (4 × 15 min) and with 1 × TBS with 0.1%TWEEN 20 (3 × 5 min). The membrane was further treated according to the AEC Staining Kit manual (AEC101-1KT, Sigma, Missouri, USA) and the positive bands became visible within 5–10 min.

### Enzyme activity analysis

The xylanase activity analysis was carried out using 2% (*w*/*v*) Azo-xylan (Megazyme, Bray, IE) as the substrate in McIlvaine buffer (pH 7) according to the modified assay as described (Busk and Lange 2013b). First, 20 μl of enzyme supernatant was mixed with 20 μl of substrate and incubated at 50 °C for 1 h. Next, 100 μl precipitant (300 mM CH_3_COONa, 18 mM (CH_3_CO_2_)_2_Zn, 76% ethanol (pH 5)) was added and vortexed, incubated at room temperature for 10 min, followed by vortexing again, and then centrifuged at 16000*g* for 1 min. Lastly, 100 μl of the supernatant was transferred to a 96-well plate and the absorbance was measured at 600 nm.

The β-xylosidase activity was detected using 5 mM 4-nitrophenyl-β-d-xylopyranoside (*p*NPX) (Sigma, Copenhagen, DK) as the substrate in McIlvaine buffer (pH 7) as described (Huang et al. 2015). First, 15 μl purified enzyme was added to 150 μl substrate and incubated at 25 °C for 10 min. Then, 30 μl of the reaction sample was transferred to a 96-well plate and the reaction was terminated by adding 50 μl 1 M Na_2_CO_3_. The absorbance was measured at 405 nm. One unit of β-xylosidase activity was defined as the amount of enzyme required to produce 1 μmol *p*-nitrophenol (*p*NP) per minute under the described assay conditions.

### Effects of temperature and pH on the enzyme activity

The optimum reaction pH of purified RrXyn11A and RrXyl43A was investigated as described above except that the reactions were conducted in McIlvaine buffer at different pH values. The pH stability of purified enzymes was analyzed by incubating the enzyme at different pH (McIlvaine buffer pH 2, 3, 4, 5, 6, 7, and 8, and 50 mM sodium carbonate buffer pH 9, 10, and 11) at 4 °C for 1 h. Residual activity was detected as described above. The optimum reaction temperature of purified RrXyn11A and RrXyl43A was determined as described above except that the reactions were incubated at different temperatures (25, 40, 50, 60, 70 °C). The thermostability of enzyme was also analyzed by incubating the purified enzymes for 1 h at 25, 40, 50, 60, and 70 °C in McIlvaine buffer (pH 7), and residual enzyme activity was measured as described above.

### Protein determination

The protein concentration of the purified recombinant enzymes and commercial enzymes was determined with the Pierce™ BCA Protein Assay Kit (23225, Thermo Scientific, Rockford, USA) and BSA used as standards.

### Substrate specificity

*p*NPX, 4-nitrophenyl-α-l-arabinofuranoside (*p*NPA) (Sigma, Copenhagen, DK) and 4-nitrophenyl-β-d-glucopyranoside (*p*NPG) (Sigma, Copenhagen, DK), azurine-cross-linked (AZCL) arabinoxylan, AZCL xylan oat, AZCL debrancharabinan, AZCL HE-cellulose, AZCL xyloglucan, and AZCL barley-glucan (Megazyme, Bray, IE) were used as substrates to test the enzyme-specific activity. When using the *p*NPX, *p*NPA, and *p*NPG as substrates, the reaction and unit calculation was performed as described above (“Enzyme activity analysis” section). The assay using the AZCL substrates was performed as described by Huang et al. (2015). The enzyme activity was indicated by the diameter of blue haloes measured as millimeters.

### Xylose tolerance of the recombinant RrXyl43A

Reactions were carried out at xylose concentrations ranging from 0 to 150 mM to investigate the xylose tolerance of the purified recombinant RrXyl43A. The residual activity was measured as described above (“Enzyme activity analysis” section).

### Kinetic properties

The Michaelis–Menten kinetics of the purified recombinant RrXyn11A and RrXyl43A were determined using different concentrations of beechwood xylan (1–25 mg/ml) and *p*NPX (0.1–10 mM) in McIlvaine buffer (pH 7), respectively. The enzyme reactions were measured as described below when using beechwood xylan as substrate, but with an incubation time of only 10 min, and as described below (see “Enzyme activity analysis” section) when using *p*NPX as substrate. One unit of xylanase activity was defined as the amount of enzyme required to produce 1 mmol xylose per minute under the described assay conditions. The data were calculated by the enzyme kinetics Michaelis–Menten method using a nonlinear regression (curve fit) program GraphPad Prism 7.03 (https://www.graphpad.com/).

### Hydrolysis of wheat bran, corn bran, and beechwood xylan and analysis of the products

Wheat bran and corn bran were destarched by treatment with 0.1% (mg protein/mg substrate) amylase (Termamyl 120 L, Sigma, Copenhagen, DK) for 2 h at 85 °C and pH 6, followed by treatment with 0.1% (mg protein/mg substrate) amyloglucosidase (AMG 300 L, Sigma, Copenhagen, DK) for 2 h at 60 °C, pH 4.5. The destarched wheat bran and corn bran were freeze-dried and used as substrate for enzymatic hydrolysis. The enzymatic hydrolysis was carried out in a suspension containing 0.01 g/ml beechwood xylan, destarched wheat bran, or destarched corn bran, and 0.1% (mg enzyme protein/mg substrate) xylanase, or a combination of xylanase and xylosidase, in 1.2 ml McIlvaine buffer at pH 7. Commercial xylanase Pulmozyme HC (Novozyme, Bagsvaerd, DK) and β-1,4-xylosidase (Megazyme, Bray, IE) were used as positive controls. Buffer was added instead of enzymes as negative control. Reactions were carried out in a thermo-shaker at 1000 rpm at 40 °C for 24 h. The reactions were terminated by incubating at 99 °C for 10 min. The mixtures were centrifuged at 16000*g* for 5 min and the supernatant was filtered before further analysis.

The endo-activity of the xylanase RrXyn11A was determined by measuring the reducing sugars according to Lever (1977) with modifications. A 10-μl sample was added to 290 μl of PAHBAH solution (0.5% *w*/*v* of PAHBAH (4-hydroxybenzoic acid hydrazide), bismuth nitrate, and potassium-sodium-tartrate in 0.5 M NaOH), mixed well and incubated at 100 °C for 5 min. The reactions were cooled to room temperature before measuring the reducing ends at 410 nm. d-xylose was included as standard.

Xylo-oligosaccharides were analyzed by high-performance anion exchange chromatography with pulsed amperometric detection (HPAEC-PAD) carried out on a Dionex ICS3000 system (Dionex Corp., Sunnyvale, CA) using a CarboPac PA1 (4 mm × 250 mm) analytical column (Thermo Fisher Scientific, Waltham, USA) equipped with a CarboPac PA1 (4 mm × 50 mm) guard column (Dionex Corp., Sunnyvale, CA). The eluent system comprised MilliQ water (A), 500 mM NaOH (B), and 500 mM NaOAc with 0.02% (*w*/*v*) NaN3 (C); a flow rate of 1 ml/min; and 10 μl injection volume. Compounds were eluted isocratically with 60:40:0 (%A:B:C) for 2 min followed by a linear gradient up to 15:40:45 (%A:B:C) over 33 min. The column was cleaned and re-equilibrated with 5:5:90 (%A:B:C) for 4 min and 60:40:0 (%A:B:C) for 6 min, respectively. Quantification was performed using external standards of xylose and linear xylo-oligosaccharides DP 2-6 (Megazyme, Bray, IE).

### Statistical analysis

Statistical analysis was performed using JMP® version 13.0.0 (SAS Institute Inc., Cary, NC). One-way ANOVA and Tukey’s multiple-comparison test were used to determine significant differences among measured reducing ends, released xylose and xylo-oligosaccharides. Tests were considered to be statistically significant if *P* values lower than 0.05 were obtained.

## Results

### Sequence properties of the predicted RrXyn11A and RrXyl43A from *R. rosea* genome

The function score from the HotPep function prediction of RrXyn11A indicated that this enzyme has mainly endo-1,4-β-xylanase activity (EC 3.2.1.8) and that RrXyl43A has specific1,4-β-xylosidase activity (EC 3.2.1.37) (Table 1). The functions of RrXyn11A and RrXyl43A were supported further by dbCAN analysis and confirmed by the following activity analysis. The sequence of RrXyn11A has a predicted signal peptide, a GH11 and a CBM1 domain. The RrXyl43A sequence has a GH43 subfamily 1 domain and no predicted signal peptide. According to the Euk-mPLoc 2.0 prediction (http://www.csbio.sjtu.edu.cn/bioinf/euk-multi/), the native protein is most likely transported to the cytoplasm. The predicted *N*-glycosylation sites of RrXyn11A were N25, N38, and N230 with 0.7257, 0.6413, and 0.6532 potential, respectively. There was no predicted *N*-glycosylation site for RrXyl43A.

**Table 1:** Sequence characterization of the HotPep-predicted RrXyn11A and RrXyl43A in *R. rosea* genome

Sequence ID	PPR/HotPep information			Predicted function	EC number	Cellular localization prediction		dbCAN	*N*-glycosylation
	Function score	Length	ORF hits			SignalP	Euk-mPLoc 2.0		
RrXyn11A	3.2.1.8(1430), 3.1.1.72(27), 3.2.1.32(3), 3.2.1.73(1)	290	64	endo-1,4-β-xylanase	3.2.1.8	Y (position 1–19)	Extracell.	Glycosyl hydrolase family 11 (position 18–193) + CBM1 (position 232–260)	N25, N38, and N230
RrXyl43A	3.2.1.37(243), 3.2.1.99(0), 3.2.1.55(0)	325	48	1,4-β-xylosidase	3.2.1.37	N	Cytoplasm. extracell.	GH43 subfamily 1 (position 7–319)	N

**Fig. 1:**
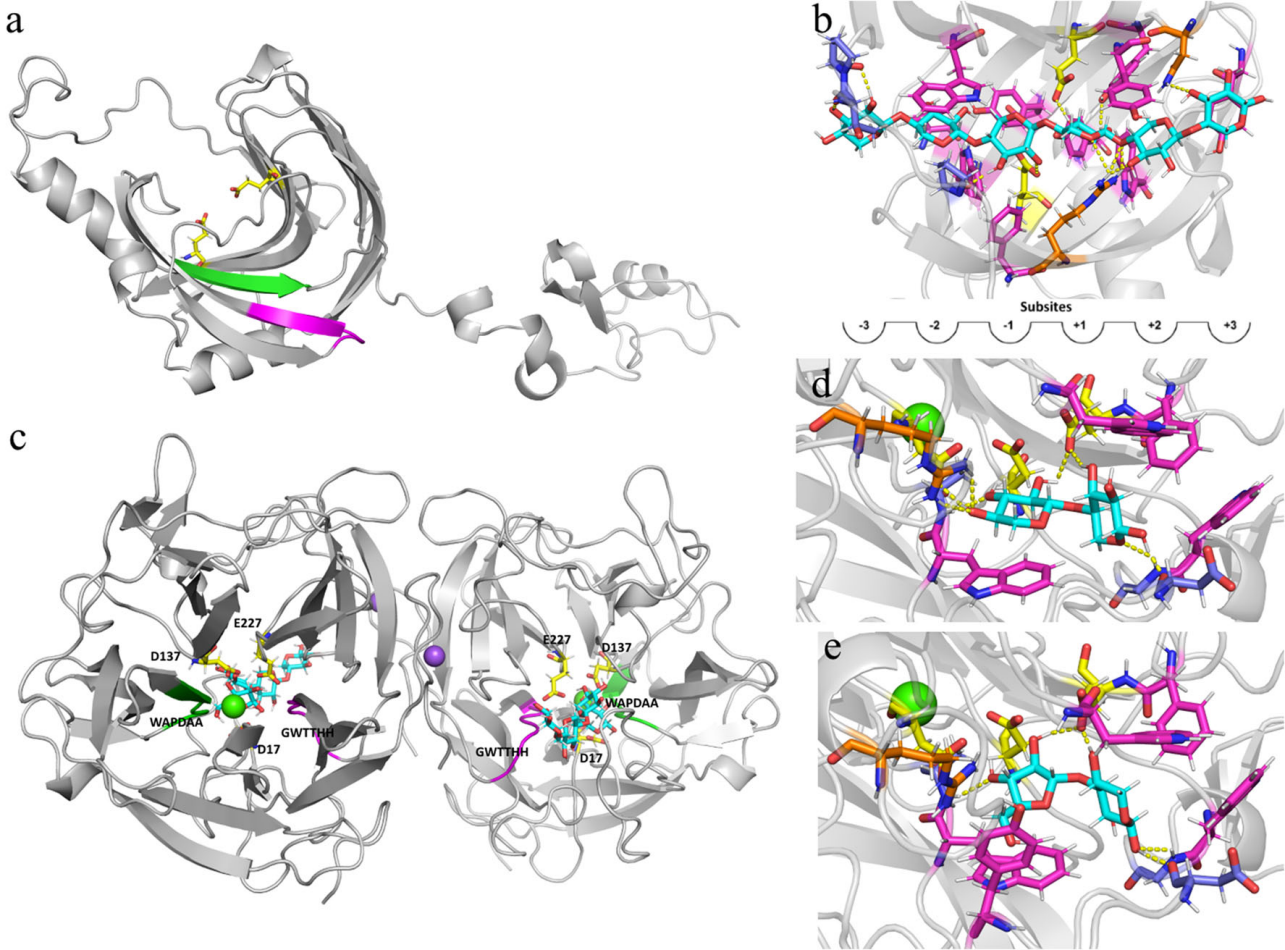
3D model structure analyses of xylanase and xylosidase from *Rhizophlyctis rosea*. 3D model structure of RrXyn11A (**a**) and a xylohexaose superimposed structure of RrXyn11A (**b**); 3D model structure of RrXyl43A (**c**), a xylobiose superimposed structure (**d**), and a Ara-(α1-3)Xyl superimposed structure of RrXyl43A (**e**). The amino acids of the active site are highlighted as yellow sticks. The peptides with highest frequency from HotPep analysis (RrXyn11A: DGGTYD, and RrXyl43A: GWTTHH) are highlighted in pink. The peptides with second highest frequency from HotPep analysis (RrXyn11A: QYWSVR, and RrXyl43A: WAPDAA) are highlighted in green. Ca^2+^ and Na^+^ ions are shown as green and purple spheres, respectively. Modeled or superimposed ligands are shown as cyan sticks. Residues involved in substrate binding are highlighted as sticks with the following colors: aromatic residues in magenta, protein backbone in purple, and residue sidechains in orange. Polar interactions between substrate and protein are indicated as yellow dotted lines

The 3D modeling structures of RrXyn11A and RrXyl43A can be found in Fig. 1. RrXyn11A showed the typical β-jelly-roll catalytic domain connected to a CBM1 domain. The overall QMEAN4 *Z*-score of 0.49 indicated model quality was good, though the N terminus and the CBM1 had somewhat lower scores due to their flexibility. The active site residues E118 and E209 and the HotPep hexamers DGGTYD and QYWSVR with high frequency were in the catalytic cleft of RrXyn11A (Fig. 1a). The substrate superimposed model can be found in Fig. 1b. As expected for carbohydrate-active enzymes, the substrate is bound by several aromatic amino acid residues that align the subsites. The substrate binding in the model is additionally supported by polar interactions of other residue side chains and the protein backbone. The positioning of the xylohexaose clearly indicates the endo-xylanase activity of the enzyme. In the superimposed structure, two xylotrioses would be released after cleavage in the middle of the substrate. The 3D model of RrXyl43A showed the typical fivefold β-propeller structure and was modeled as a dimer where both monomers are involved in the binding of two Na^+^ ions (Fig. 1c). A Ca^2+^ ion was modeled close to the active site though the exact function of this is not fully clear. The overall QMEAN4 Z-score of 0.23 indicated that this model also had good quality. Lastly, the program also modeled two products into the structure, arabinose and xylotriose, which could be used for superposition of putative substrates (Fig. 1d, e). Both superimposed substrates, xylobiose and Ara-(α1 → 3)-Xyl, fitted well in the active site and were coordinated by mainly aromatic amino acid side chains and polar protein backbone interactions. However, the xylobiose is additionally coordinated by an Arg sidechain (R305, Fig. 1d), which was not found in the AX-bound model (Fig. 1d).

**Fig. 2:**
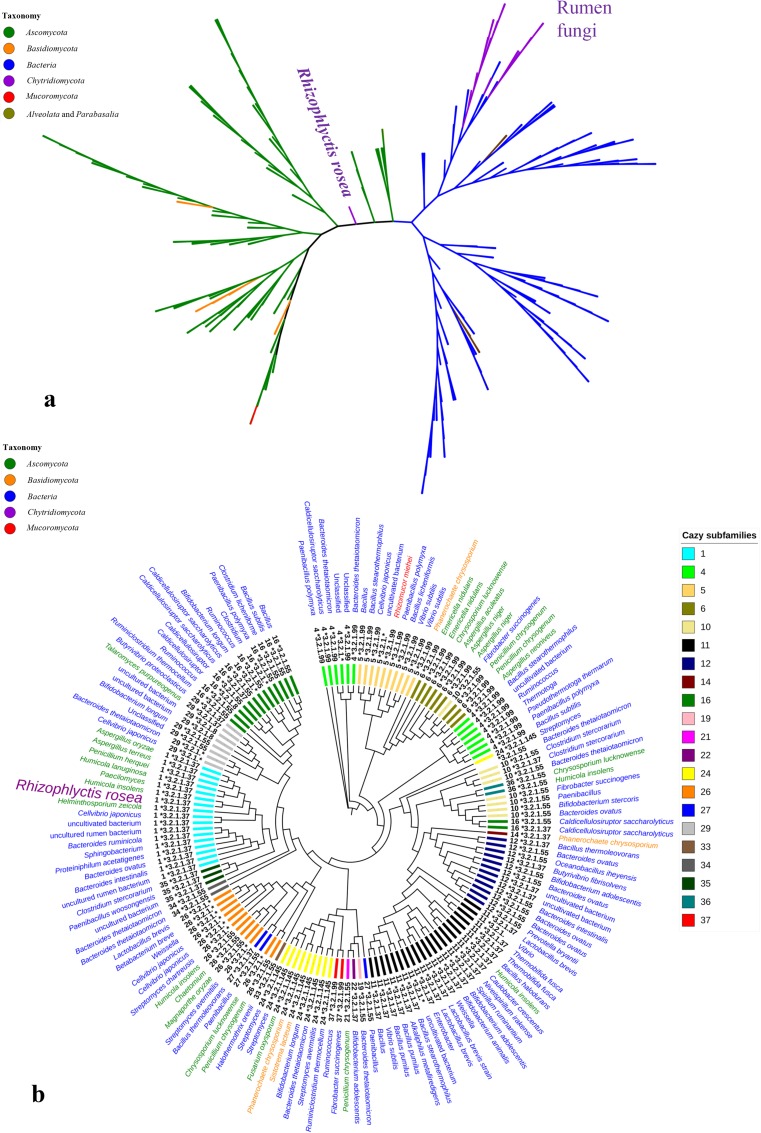
Phylogenic analyses of GH11 xylanases and GH43 xylosidases. **a** Radial tree of GH11 xylanases including RrXyn11A from *Rhizophlyctis rosea*; **b** circular tree of GH43 xylosidases including RrXyl43A from *Rhizophlyctis rosea*. The outer ring is the taxonomy of the origins of each sequence (taxonomy color legend (left)); the inner ring is the CAZy subfamilies (subfamily color legend (right)) and EC number and CAZy subfamily number (**b**) of each sequence. The accession numbers of the sequences which were selected for the phylogenetic tree can be found in Supplemental Fig. S1 and S2

The phylogenetic tree of GH11 xylanases showed that the xylanases from fungi (*Ascomycota*, *Basidiomycota*, and *Mucoromycota*) were separated from the xylanases from bacteria. However, xylanases from anaerobic early-lineage fungi (*Neocallimastix* sp., *Orpinomyces* sp., and *Piromyces* sp.) from rumen of herbivorous animals were grouped together with the xylanases from bacteria. RrXyn11A from *R. rosea* was separated from the enzymes from ascomycetous fungi and was also separate from the enzymes from bacteria and rumen chytrids (Fig. 2a). The phylogenetic tree of the sequences from different GH43 CAZy subfamilies indicated that RrXyl43A from *R. rosea* was in GH43 subfamily 1 which includes enzymes from both bacteria and fungi. The sequence closest to RrXyl43A was from the thermophilic ascomycetous fungus, *H. insolens* (Fig. 2b).

### Heterologous expression of RrXyn11A and RrXyl43A in *P. pastoris*

**Fig. 3:**
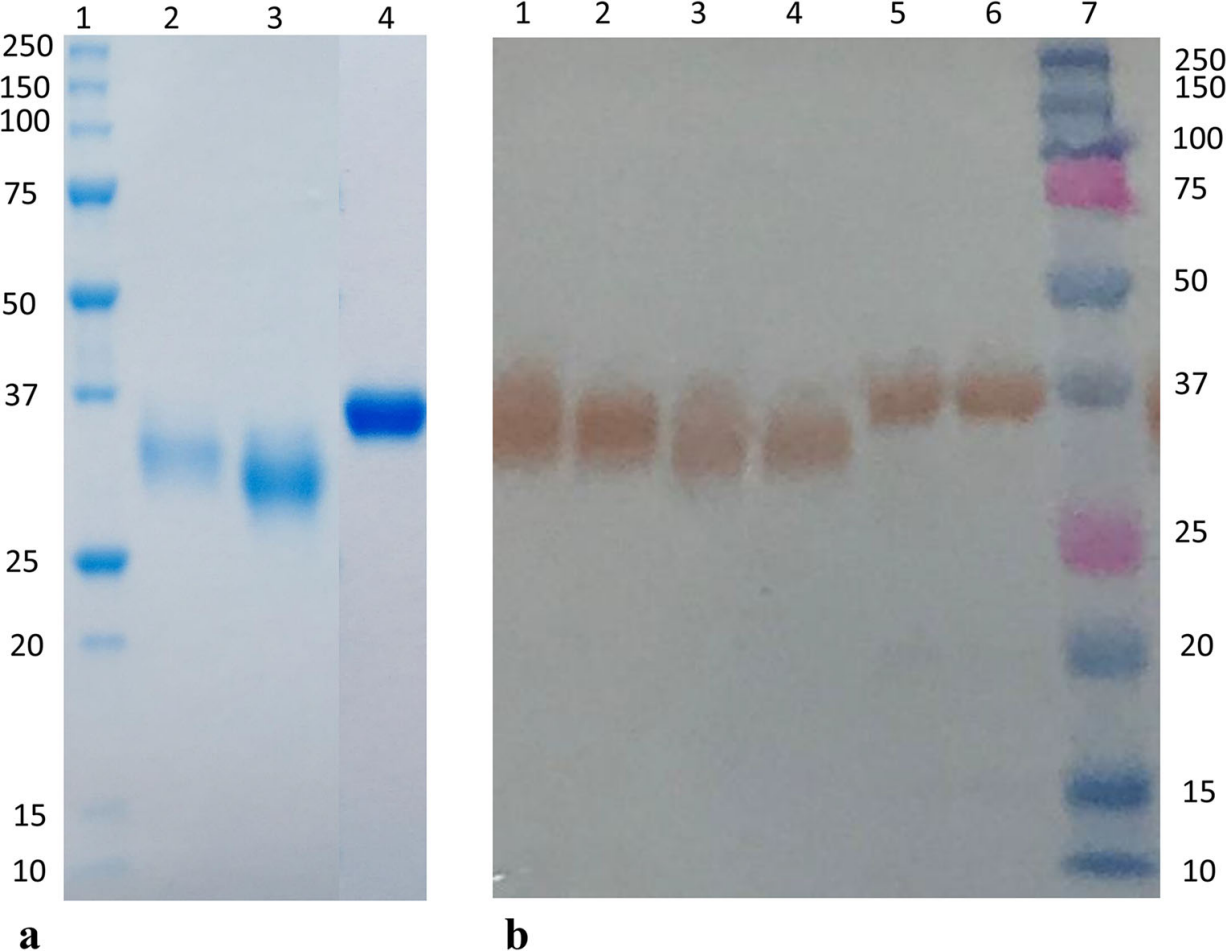
SDS-PAGE and western blot analysis of purified recombinant RrXyn11A and RrXyl43A**. a** SDS-PAGE, lane 1: Precision Plus Protein™ Unstained Protein Standards; lane 2: RrXyn11A L (larger molecular weight); lane 3: RrXyn11A S (smaller molecular weight); and lane 4: RrXyl43A. **b** Western blot, lane 1: RrXyn11A L; lane 2: Endo H treated RrXyn11A L; lane 3: RrXyn11A S; lane 4: Endo H treated RrXyn11A S; lane 5: RrXyl43A; lane 6: Endo H treated RrXyl43A; lane 7: Precision Plus Protein™ Dual Color Standards

Xylanase RrXyn11A and xylosidase RrXyl43A were successfully expressed in *P. pastoris* KM71H. The theoretical molecular weight of the recombinant RrXyn11A and RrXyl43A with His tag was 31 and 39 kDa, respectively. The purification of the recombinant RrXyn11A produced two peaks, and the fractions for each peak were collected and defined as RrXyn11A S (smaller molecular weight) and RrXyn11A L (larger molecular weight) (Fig. 3a). The treatment of recombinant enzymes with Endo H suggests that the recombinant RrXyn11A and RrXyl43A were not *N*-glycosylated, since no change in molecular weight was observed (Fig. 3b). Both fractions of RrXyn11A S and RrXyn11A L were detected by western blot using anti-His tag antibody (Fig. 3b) and exhibited xylanase activity.

### Effects of temperature and pH on recombinant RrXyn11A and RrXyl43A activity and stability

**Fig. 4:**
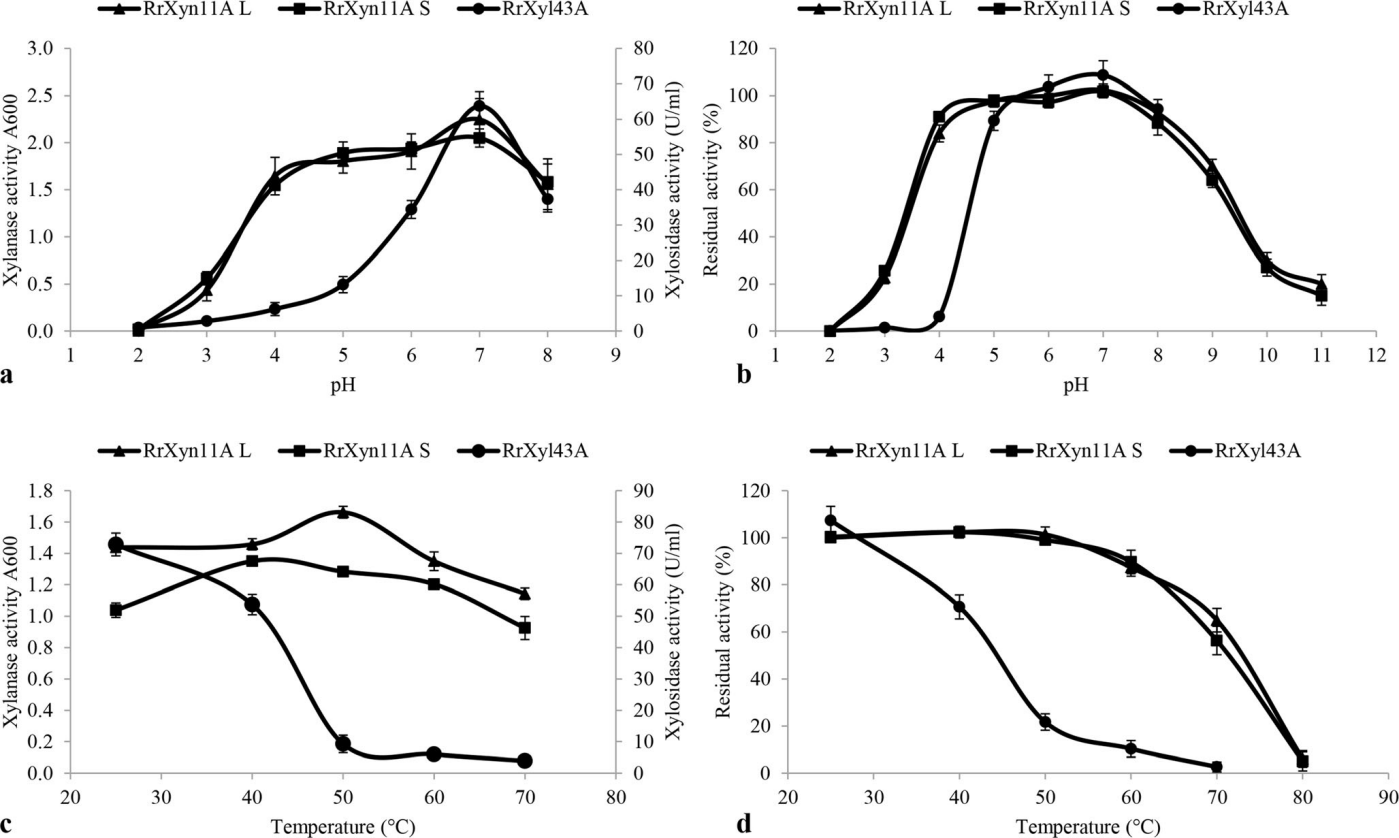
Effects of temperature and pH on recombinant RrXyn11A and RrXyl43A activity and stability. **a** pH profile of RrXyn11A L, RrXyn11A S, and RrXyl43A; **b** pH stability of RrXyn11A L, RrXyn11A S, and RrXyl43A; **c** temperature profile of RrXyn11A L, RrXyn11A S, and RrXyl43A; **d** thermostability of RrXyn11A L, RrXyn11A S, and RrXyl43A

The optimal pH for RrXyn11A S, RrXyn11A L, and RrXyl43A was pH 7 (Fig. 4a). RrXyn11A S and RrXyn11A L were stable over pH 4–8 and RrXyl43A was stable over pH 5–8 (Fig. 4b). The optimal reaction temperature for RrXyn11A S, RrXyn11A L, and RrXyl43A was 40, 50, and 25 °C, respectively (Fig. 4c). The thermostability results showed that the residual activity of RrXyn11A S and RrXyn11A L after incubation at 70 °C for 1 h was 56% and 65%, respectively. The residual activity of RrXyl43A was still 72% after incubation at 40 °C for 1 h (Fig. 4d).

### Substrate specificity and kinetic analysis of recombinant RrXyn11A and RrXyl43A

In this study, eight substrates were used to investigate the substrate specificity of the purified recombinant enzymes (Table 2). The results showed that RrXyn11A S and RrXyn11A L were active only on AZCL arabinoxylan and AZCL xylan oat substrates, and showed blue haloes which indicated that both of them have only endo-1,4-β-d-xylanase activity (EC 3.2.1.8). RrXyl43A had not only β-xylosidase activity but also low α-l-arabinofuranosidase activity. The kinetic parameters of xylanases RrXyn11A S and RrXyn11A L and of xylosidase RrXyl43A were determined using, respectively, beechwood xylan and 4-nitrophenyl-β-d-xylopyranoside (*p*NPX) as the substrate. The *K*_m_ for RrXyn11A S, RrXyn11A L, and RrXyl43A was 21.63 mg/ml, 18.83 mg/ml and 1.34 mM, respectively. The *V*_max_ for RrXyn11A S, RrXyn11A L, and RrXyl43A was 219.5, 210.7, and 34.61 U/mg, respectively (Table 3 and Supplemental Fig. S3).

**Table 2:** Substrate specificity for purified recombinant RrXyn11A and RrXyl43A

Substrate	Activity type (activity unit)	RrXyn11A L	RrXyn11A S	RrXyl43A
4-Nitrophenyl-β-d-xylopyranoside	β-xylosidase (U/mg)	–	–	36.66
4-Nitrophenyl-α-l-arabinofuranoside	α-l-arabinofuranosidase (U/mg)	–	–	3.41
4-Nitrophenyl-β-d-glucopyranoside	β-glucosidase (U/mg)	–	–	–
AZCL arabinoxylan	Endo-1,4-β-d-xylanase (mm)	18	20	–
AZCL xylan oat	Endo-1,4-β-d-xylanase (mm)	18	20	–
AZCL debrancharabinan	Endo-1,5-α-l-arabinanase (mm)	–	–	–
AZCL HE-cellulose	Endo-β-1,4-glucanase (mm)	–	–	–
AZCL xyloglucan	Endo-β-1,4-xyloglucanase (mm)	–	–	–
AZCL barley-glucan	Endo-β-1,3-1,4-glucanase (mm)	–	–	–

**Table 3:** Kinetic parameters of purified recombinant RrXyn11A and RrXyl43A

Recombinant enzyme	Substrate	Reaction condition	*K*_m_*	*V*_max_ (U/mg)	*k*_cat_ (s^−1^)
RrXyn11A S	beechwood xylan	pH 7, 40 °C, 10 min	21.63 ± 3.43	219.5	106.25 ± 10.53
RrXyn11A L	beechwood xylan	pH 7, 50 °C, 10 min	18.83 ± 3.56	210.7	54.80 ± 6.21
RrXyl43A	*p*NPX	pH 7, 25 °C, 10 min	1.34 ± 0.17	34.61	21.20 ± 0.89

*The *K*_m_ unit of RrXyn11A L and RrXyn11A S is mg/ml; the *K*_m_ unit of RrXyl43A is mM

### Xylose tolerance of RrXyl43A

The xylose tolerance of RrXyl43A was investigated at different xylose concentrations (0–150 mM) using *p*NPX as substrate. The RrXyl43A activity was stable at xylose concentration below 10 mM. When the xylose concentration was increased above 100 mM, RrXyl43A retained 51% activity (Supplemental Fig. S4).

### Enzymatic hydrolysis of beechwood xylan, wheat bran, and corn bran

**Fig. 5:**
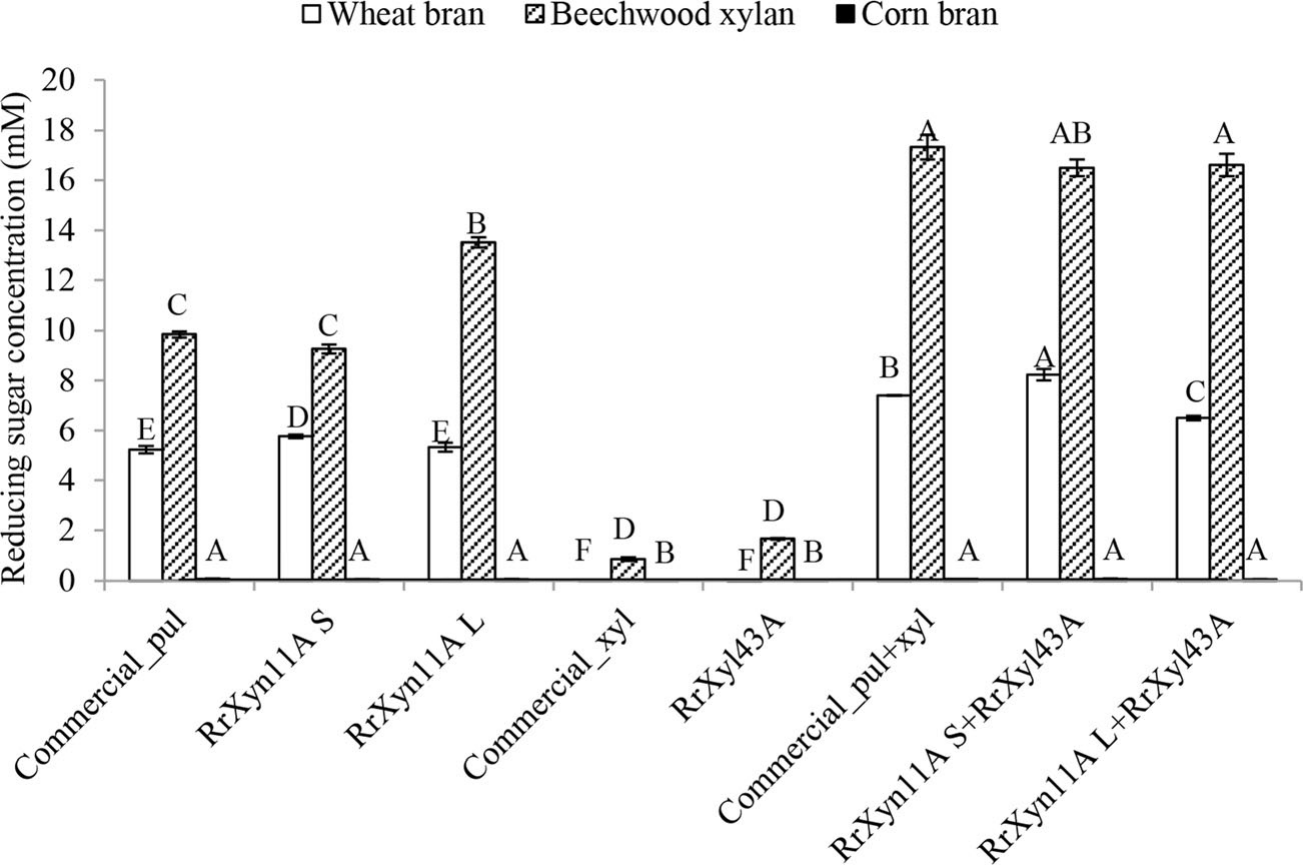
Reducing sugar after enzyme hydrolysis of beechwood xylan, wheat bran, and corn bran. commercial_pul, commercial Pulmozyme HC. commercial_xyl, commercial 1.4-β-D-xylosidase. Statistical significance is indicated by capital letters

In this study, the purified recombinant RrXyn11A and RrXyl43A have been further applied for hydrolysis of beechwood xylan, wheat bran, and corn bran. The commercial enzymes Pulmozyme HC (Novozyme, Bagsvaerd, DK) and 1,4-β-d-xylosidase (Megazyme, Bray, IE) were used as positive controls. When wheat bran was used as substrate, the hydrolytic activity of RrXyn11A S (measured as reducing sugar equivalents) was 5.77 mM, which was higher than for hydrolysis by Pulmozyme HC (5.23 mM). The combined treatment with RrXyn11A S and RrXyl43A also released significantly higher reducing sugar equivalents (8.23 mM) than the combination of commercial Pulmozyme HC and 1,4-β-d-xylosidase (7.41 mM) (Fig. 5). When beechwood xylan was used as substrate, the amount of reducing sugar equivalents released after hydrolysis by RrXyn11A S and RrXyl43A combined (16.50 mM) and by RrXyn11A L and RrXyl43A combined (16.61 mM) was close to that obtained with the commercial enzymes combined (17.32 mM) (Fig. 5).

**Fig. 6:**
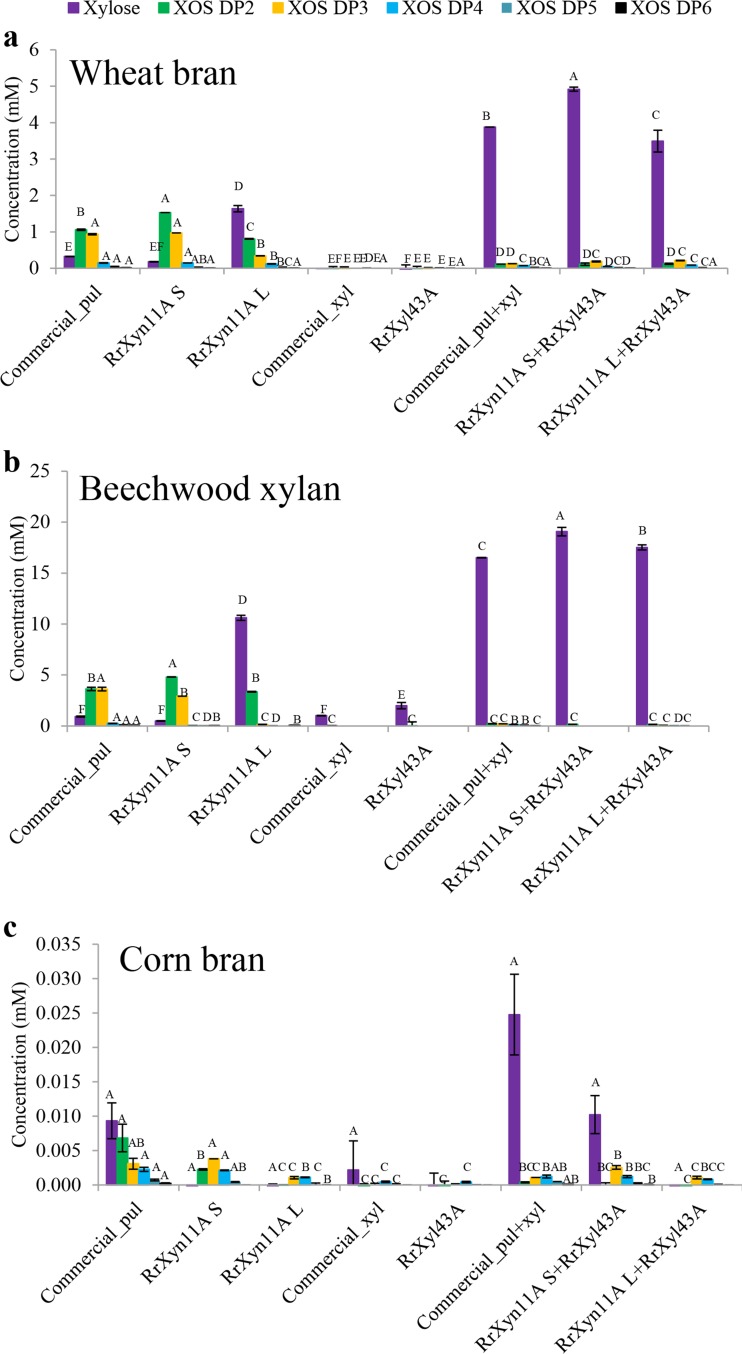
Xylo-oligosaccharide concentration after enzyme hydrolysis of different substrates. Xylo-oligosaccharide concentration after monocomponent enzyme and combination of enzymes hydrolysis of wheat bran (**a**), beechwood xylan (**b**), and corn bran (**c**). commercial_pul, commercial Pulmozyme HC (Novozyme, Bagsvaerd, DK). commercial_xyl, commercial 1,4-β-d-xylosidase (Megazyme, Bray, IE). Statistical significance is indicated by capital letters

When the commercial enzyme Pulmozyme HC and recombinant RrXyn11A S were applied individually to hydrolyze wheat bran and beechwood xylan, they released higher concentrations of XOS2 and XOS3, followed by xylose and XOS4, XOS5, and XOS6 (Fig. 6a, b). However, when xylanase RrXyn11A L was use alone, the reaction released much higher concentrations of xylose than XOS2 and XOS3. Neither the commercial 1,4-β-d-xylosidase nor the recombinant RrXyl43A showed any activity on wheat bran, and only very low amounts of xylose and XOS2 could be detected with beechwood xylan as the substrate (Fig. 6a, b). When wheat bran was used as substrate, the combination of RrXyn11A S and RrXyl43A released significantly more xylose (4.92 mM) than the combination of commercial enzymes (3.88 mM), which in turn released more xylose than RrXyn11A L and RrXyl43A combined (3.49 mM) (Fig. 6a). However, when beechwood xylan was used as substrate, the amount of xylose released after hydrolysis by the above two combinations of recombinant enzymes (19.07 and 17.52 mM, respectively) was much higher than with the commercial enzymes (16.52 mM) (Fig. 6b). The reducing sugar and xylo-oligosaccharides obtained for both recombinant and commercial enzyme treatments of corn bran were much lower than when wheat bran and beechwood xylan were the substrates (Figs. 5 and 6c).

## Discussion

*R. rosea* has been reported as a cellulose decomposer which can be easily recovered from fertile soil (Chambers and Willoughby 1964; Willoughby 2001). Lange et al. (2018) found that *R. rosea* has a high number of carbohydrate-active enzymes encoded in the genome and found in the secretome when it was grown in the medium with Avicel, CMC (carboxymethylcellulose), glucose, and wheat bran as carbon source. However, until now, only one endoglucanase from the GH45 family has been heterologously expressed and characterized from this fungus (Pilgaard 2014; Lange et al. 2018). This study is the first to describe and characterize the recombinant xylan-degrading enzymes RrXyn11A (EC 3.2.1.8) and RrXyl43A (EC 3.2.1.37) from the chytrid *R. rosea*.

3D modeling indicated that the HotPep-predicted hexamers with high frequency of RrXyn11A were close to the active sites in the catalytic pocket. Although one of the two HotPep-predicted hexamers (QYWSVR) was involved in substrate binding for RrXyn11A, both seem to be more generally involved in formation of the overall β-jelly roll fold. However, for RrXyl43A, both HotPep-predicted hexamers are involved in binding the Ca^2+^ ion, and one (WAPDAA) is also involved in substrate binding. It is interesting that RrXyn11A has a CBM1 domain. Among characterized GH11 xylanases, only a few such as XYNC and XynS20 from *Neocallimastix patriciarum*, XYNB from *Penicillium funiculosum*, and XynB from *Phanerochaete chrysosporium* have a CBM1 domain (Paës et al. 2012). The supplement of PaXyn11A with CBM1 in the *Trichoderma reesei* industrial cocktail (E508) has been reported to improve the hydrolysis of wheat straw (Couturier et al. 2011). Therefore, the CBM1 domain in RrXyn11A may further improve its catalytic activity. Although the structure of RrXyl43A was modeled as a dimer (Fig. 1b), it was found to be active as a monomer. However, this is not an uncommon observation. The presence of the Ca^2+^ ion was more intriguing, and we were unaware of this during the experimental characterization of the enzyme. In fact, during preparation of this manuscript, the crystal structure of a metagenomic GH43 β-xylosidase/α-l-arabinofuranosidase which indeed is activated by Ca^2+^ was published (Matsuzawa et al. 2017). Furthermore, this might also be an explanation for the lower thermostability of RrXyl43A compared to RrXyn11A (Fig. 4d). Detailed characterization of RrXyl43A in the presence of Ca^2+^ is part of ongoing research.

It is interesting to find that RrXyn11A in the GH11 phylogenetic tree was separate from xylanases of most *Ascomycota* and was also distinct from those from rumen chytrids and bacteria. This finding indicates that RrXyn11A from *R. rosea* may have a different subspecificity regarding xylan, or simply that *R. rosea* holds an unique evolutionary position. The phylogenetic tree of GH43 enzymes indicates that RrXyl43A belongs to the GH43 subfamily 1 (EC 3.2.1.37) (Mewis et al. 2016), and the RrXyl43A protein sequence might share a close relationship with the xylosidase from thermophilic fungus *H. insolens*. Yang et al. (2014) found that the GH43 xylosidase from *H. insolens* had significant synergistic effects on the degradation of various xylans.

In this study, the xylan-degrading enzymes RrXyn11A and RrXyl43A were both successfully expressed in *P. pastoris* KM71H. RrXyl43A, without predicted signal peptide constructed with α-factor signal peptide, was expressed with a high yield of secreted protein. The purified recombinant RrXyn11A was expressed as two different proteins with different molecular weight, but both showed xylanase activity. After deglycosylation, no change in the molecular weight mass of the recombinant RrXyn11A was observed. The occurrence of enzymes with two different molecular weights might be due to *O*-glycosylation or to the unprocessed enzyme still carrying the α-factor signal sequence due to cell lysis. The optimal pH and temperature of RrXyn11A S and RrXyn11A L are similar to those of other fungi (Huang et al. 2015). However, RrXyn11A L retained 65% residual activity after 1 h incubation at 70 °C. The thermostability of this enzyme was much higher than the xylanases from mesophilic fungi such as *Fusarium* sp., *Penicillium* sp., and *T. reesei* (Huang et al. 2015; Liao et al. 2015; Tang et al. 2017) and was similar to that of thermostable GH11 xylanase from *Thermobifida halotolerans* (Zhang et al. 2012). Even though the *K*_m_ and *V*_max_ of RrXyn11A (with beechwood xylan as substrate) from *R. rosea* did not indicate the same enzyme efficiency as xylanases from *P. oxalicum* GZ-2 (Liao et al. 2015), the recombinant RrXyn11A with its high thermostability and broad pH stability could be interesting for biomass degradation (Han et al. 2017). The recombinant RrXyn11A showed high substrate specificity on arabinoxylan and oat xylan and similarities to other GH11 “true xylanases” with great potential for industrial applications and promising features for future other uses such as in food and feed technology, the fiber and paper industries, soap technology, and biofuels (Paës et al. 2012). The recombinant RrXyl43A also has minor α-l-arabinofuranosidase activity (10.75 times lower than β-xylosidase activity) which was similar to the xylosidase from *Weissella* sp. and *Bifidobacterium animalis* (Viborg et al. 2013; Falck et al. 2015) since the conformations of both substrates are sterically similar near the glycosidic bond (Mewis et al. 2016). However, the probably stronger binding of xylobiose due to a polar interaction with an Arg residue (Fig. 1d), which was not observed for a superimposed arabinose substrate, might explain the higher xylosidase compared to the arabinofuranosidase activity. Low product inhibition is important for industrial application of β-xylosidase. In this study, at a xylose concentration of 100 mM, RrXyl43A still retained 51% activity which is much higher than that observed for other fungal β-xylosidases (Kirikyali et al. 2014; Kirikyali and Connerton 2014). The *K*_m_ of RrXyl43A (with *p*PNX as substrate) from *R. rosea* was lower than the xylosidase from *H. insolens* (12.2 mM) (Yang et al. 2014), *Paecilomyces thermophila* (8 mM) (Juturu and Wu 2013), *Thermomyces lanuginosus* (3.9 mM) (Chen et al. 2012), and *Penicillium oxalicum* (4.05 mM) (Ye et al. 2017), but higher than the xylosidase from *A. oryzae* (0.48 mM) (Suzuki et al. 2010). The *k*_cat_ of RrXyl43A (with *p*PNX as substrate) from *R. rosea* (21.20/s) was much higher than that of xylosidases Xyl43A and Xyl43B from *H. insolens* (*k*_cat_ 5.51 and 0.035/s, respectively) (Yang et al. 2014), indicating that RrXyl43A may have high efficiency for substrate degradation.

This study further applied the recombinant RrXyn11A and RrXyl43A to hydrolyze beechwood xylan, destarched wheat bran, and corn bran. After destarching, arabinoxylans are the main polysaccharides in wheat bran and corn bran (Agger et al. 2010; Mendis et al. 2016). Arabinoxylans consist of a main backbone of β-(1,4)-linked d-xylopyranosyl residues, which are substituted with *R*-l-arabinofuranosyl residues linked to the C(O)-2 and/or C(O)-3 position. Phenolic acids can also be ester linked on the C(O)-5 position of arabinose. GH11 xylanases preferably cleave unsubstituted regions of the xylan backbone while xylosidases remove xylose monomers from the non-reducing end of xylo-oligosaccharides (Mendis et al. 2016). However, arabinoxylan from corn bran is recalcitrant and resists enzymatic degradation due to its complex structure and the effect of diferulic acids (Faulds et al. 1995; Saha et al. 1998; Agger et al. 2011). In this study, the commercial enzymes and the recombinant *R. rosea* enzymes, either as monocomponents or combined, could release only very low concentrations of reducing sugar or XOS with corn bran as substrate. However, the combination of both commercial and recombinant xylanase and xylosidase showed a synergic effect for the degradation of beechwood xylan and wheat bran. The combination of RrXyn11A S and RrXyl43A could even release significantly more xylose than was achieved by the combination of commercial Pulmozyme HC and 1,4-β-d-xylosidase when they were used for degradation of both beechwood xylan and wheat bran. The results indicate that the identified RrXyn11A and RrXyl43A from the chytrid *R. rosea* have great potential for industrial biomass conversion.

In conclusion, this study identified GH11 xylanase (RrXyn11A) and GH43 xylosidase (RrXyl43A) from the early-lineage fungus *R. rosea*. Both genes were heterologously expressed in *P. pastoris* and characterized. The optimal pH for recombinant RrXyn11A and RrXyl43A was pH 7. RrXyn11A had high stability over a wide pH and temperature range and high substrate specificity on both AZCL arabinoxylan and AZCL xylan from oats. RrXyl43A had β-xylosidase and minor α-l-arabinofuranosidase activity. The enzyme retained 51% activity in 100 mM xylose. The hydrolytic capability of the combination of RrXyn11A and RrXyl43A, when applied on wheat bran and beechwood xylan, released significantly more reducing sugar and xylose than a combination of commercial enzymes. Therefore, enzymes from *R. rosea* could be a highly interesting and as yet untapped source of enzymes for biomass degradation.

## Electronic supplementary material

ESM 1 (PDF 1305 kb)
