# Supplementary material for: Identification and characterization of GH11 xylanase and GH43 xylosidase from the chytridiomycetous fungus, Rhizophlyctis rosea
Source: Appl Microbiol Biotechnol. 2018 Nov 5;103(2):777–91. doi: 10.1007/s00253-018-9431-5 (PMC6373445; doi:10.1007/s00253-018-9431-5)
Supplement: Supplementary file 1 — (PDF 1305 kb) [file 253_2018_9431_MOESM1_ESM.pdf]

Supplementary material

Identification and characterization of GH11 xylanase and GH43 xylosidase from the chytridiomycetous fungus, *Rhizophlyctis rosea*

Applied Microbiology and Biotechnology

Yuhong Huang · Xianliang Zheng · Bo Pilgaard · Jesper Holck · Jan Muschiol · Shengying Li · Lene Lange

Y. Huang · B. Pilgaard · J. Holck · J. Muschiol · L. Lange (Corresponding author)
Department of Chemical and Biochemical Engineering, Technical University of Denmark, Building 229, Søltofts Plads, 2800, Kongens Lyngby, Denmark
Phone: +45 24432040; Email: lenl@kt.dtu.dk

X. Zheng
Department of Chemical and Biochemical Engineering, Technical University of Denmark, Building 229, Søltofts Plads, 2800, Kongens Lyngby, Denmark
Sino-Danish Center for Education and Research, Beijing, 100190, China
Present address: The National Food Institute, Technical University of Denmark, Building 201, Søltofts Plads, 2800, Kongens Lyngby, Denmark
Qingdao Institute of Bioenergy and Bioprocess Technology, Chinese Academy of sciences, Qingdao, 266101, China

S. Li
Qingdao Institute of Bioenergy and Bioprocess Technology, Chinese Academy of sciences, Qingdao, 266101, China

Supplemental figures

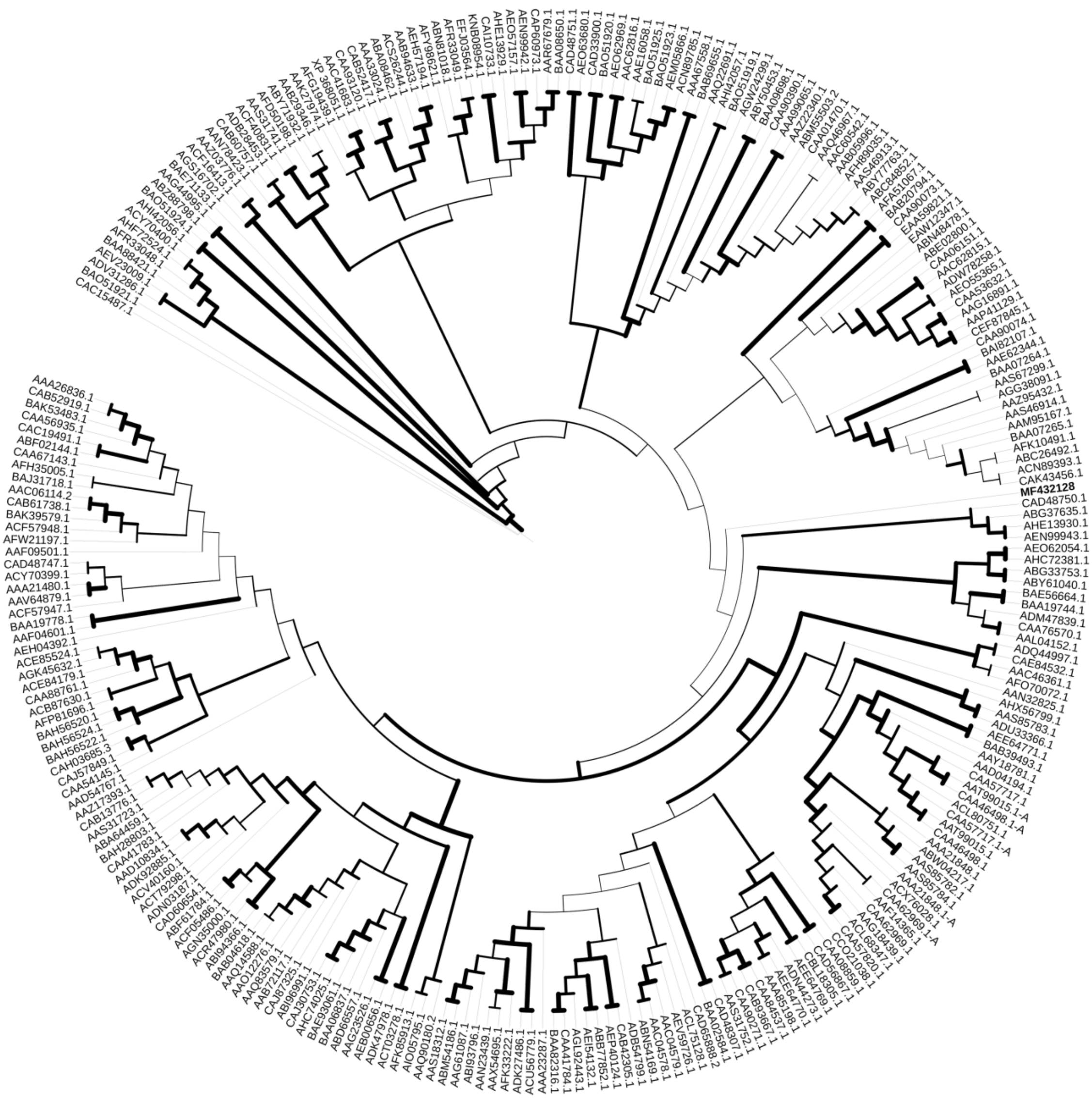

Fig. S1 Phylogenic analysis of GH11 xylanases with accession numbers of the sequences

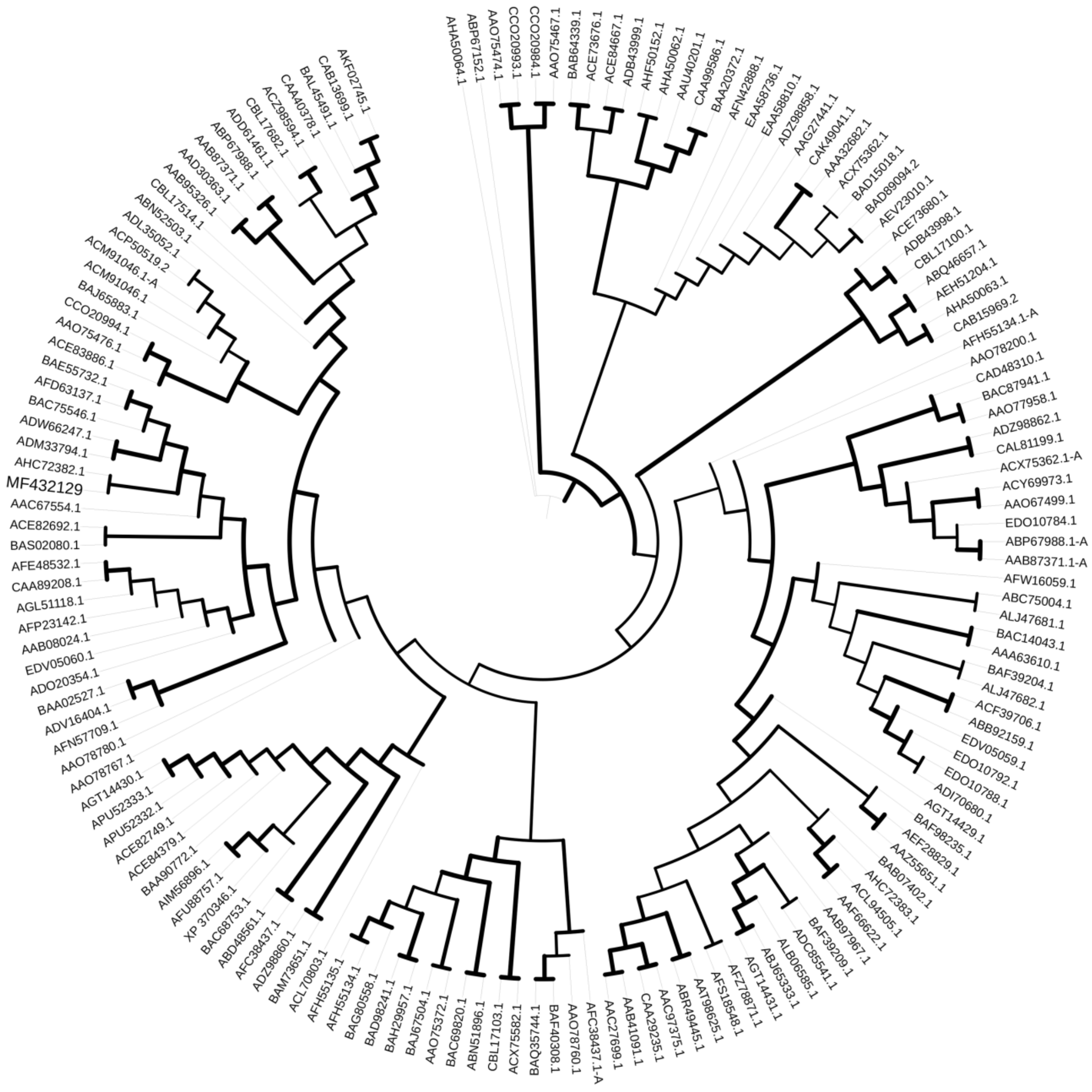

Fig. S2 Phylogenic analysis of GH43 enzymes with accession numbers of the sequences

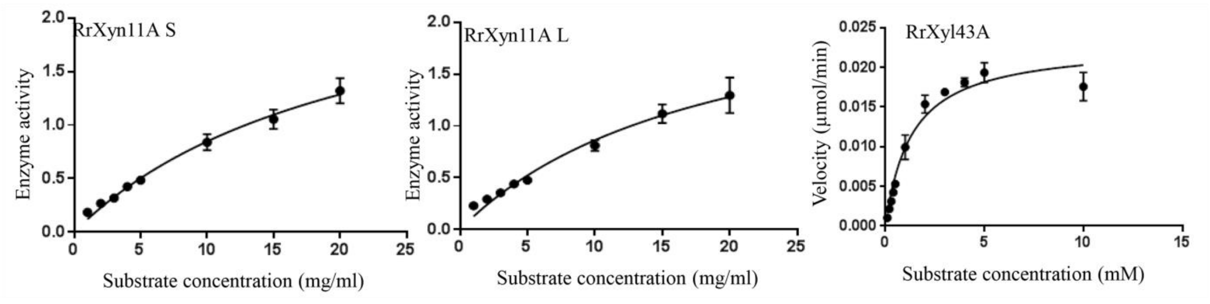

Fig. S3 Michaelis-Menten data of the recombinant RrXyn11A and RrXyl43A

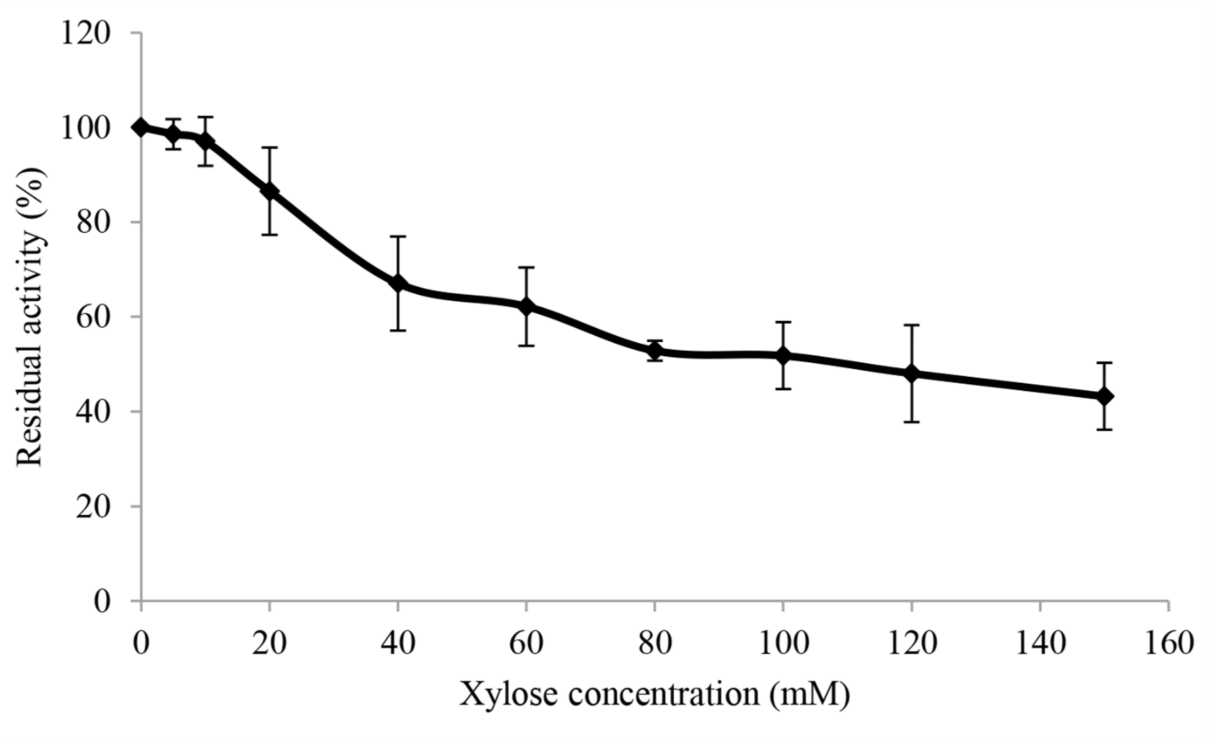

Fig. S4 Effect of xylose on the activity of the purified recombinant xylosidase RrXyl43A
